# Autophagy suppression potentiates the anti-glioblastoma effect of asparaginase *in vitro* and *in vivo*

**DOI:** 10.18632/oncotarget.19409

**Published:** 2017-07-20

**Authors:** Qicheng Chen, Li Ye, Jiajun Fan, Xuyao Zhang, Huan Wang, Siyang Liao, Ping Song, Ziyu Wang, Shaofei Wang, Yubin Li, Jingyun Luan, Yichen Wang, Wei Chen, Wenjing Zai, Ping Yang, Zhonglian Cao, Dianwen Ju

**Affiliations:** ^1^ Department of Microbiological and Biochemical Pharmacy and Key Lab of Smart Drug Delivery, Ministry of Education, School of Pharmacy, Fudan University, Shanghai, PR China; ^2^ Perelman School of Medicine, University of Pennsylvania, Philadelphia, PA, USA; ^3^ Department of Pharmacology, School of Pharmacy, Fudan University, Shanghai, PR China; ^4^ Instrumental Analysis Center, School of Pharmacy, Fudan University, Shanghai, PR China

**Keywords:** autophagy, glioblastoma, asparaginase, apoptosis, reactive oxygen species

## Abstract

Asparaginase has been reported to be effective in the treatment of various leukemia and several malignant solid cancers. However, the anti-tumor effect of asparaginase is always restricted due to complicated mechanisms. Herein, we investigated the mechanisms of how glioblastoma resisted asparaginase treatment and reported a novel approach to enhance the anti-glioblastoma effect of asparaginase. We found that asparaginase could induce growth inhibition and caspase-dependent apoptosis in U87MG/U251MG glioblastoma cells. Meanwhile, autophagy was activated as indicated by autophagosomes formation and upregulated expression of LC3-II. Importantly, abolishing autophagy using chloroquine (CQ) and LY294002 enhanced the cytotoxicity and apoptosis induced by asparaginase in U87MG/U251MG cells. Further study proved that Akt/mTOR and Erk signaling pathways participated in autophagy induction, while reactive oxygen species (ROS) served as an intracellular regulator for both cytotoxicity and autophagy in asparaginase-treated U87MG/U251MG cells. Moreover, combination treatment with autophagy inhibitor CQ significantly enhanced anti-glioblastoma efficacy of asparaginase in U87MG cell xenograft model. Taken together, our results demonstrated that inhibition of autophagy potentiated the anti-tumor effect of asparagine depletion on glioblastoma, indicating that targeting autophagy and asparagine could be a potential approach for glioblastoma treatment.

## INTRODUCTION

Glioblastoma (GBM), derived from central nervous system (CNS) cells, is the most frequent primary brain tumor in adults [1]. Currently, the standard treatment for newly diagnosed GBM is surgical resection in combination with radiotherapy and chemotherapy [2–4]. However, deep tumor infiltration of GBM cells into normal tissues results in almost impossible complete resection [5]. Besides, due to the existence of blood-brain barrier (BBB) and emergence of resistance to chemotherapeutic drug, chemotherapy is only minimally effective [6, 7]. Thus, the prognosis has remained dismal, with a 14.6-month median overall survival and a 6.9-month median progression free survival, and five-year survival rate is only 9.8% after diagnosis [3, 4, 8]. Therefore, novel therapeutic strategies are urgently needed to realize the long-term survival of GBM patients.

Targeting cancer amino acid metabolism has been applied safely and effectively in the treatment of plenty of malignancies [9–12]. Asparaginase, an enzyme drug which deprives tumor cells of semi-essential L-asparagine by hydrolyzing extracellular L-asparagine into L-aspartic acid and ammonia, has been used clinically for treating acute lymphoblastic leukemia (ALL) since early 1970s [13, 14]. Asparagine synthetase (ASNS) in eukaryotic cells is able to utilize L-aspartate and L-glutamine as a substrate to produce L-asparagine, while its expression in most malignancies is absent or low [14, 15]. Thus, L-asparagine is an essential amino acid for cancer cell growth, and asparagine depletion has become one of the most promising strategies for cancer therapy [15–17]. Except for being proved for treatment of ALL, asparaginase is also reported to be effective in treatment of other kinds of leukemia including non-Hodgkin’s lymphoma [18], acute myelocytic leukemia (AML) [19, 20], chronic myelocytic leukemia (CML) [21], chronic lymphocytic leukemia (CLL) and several malignant solid cancers such as melanosarcoma [22, 23], ovarian cancer [24, 25] and pulmonary adenocarcinoma (ADCA) [26]. Recent study reported that depletion of asparagine by asparaginase sensitized GBM cells to apoptosis *in vitro* and *in vivo* [10].

An increasing number of studies have shown that amino acid deprivation therapy, including asparagine deprivation therapy and arginine deprivation therapy, can induce autophagy in tumor cells [21, 25–29]. Autophagy is an evolutionally conserved cellular catabolic process in eukaryotic cells, and can delivery dysfunctional proteins and organelles in cytoplasm for lysosomal degradation to maintain the cellular environmental homeostasis under metabolic stress conditions [30, 31]. Autophagy is widely implicated in vital biological processes including differentiation, aging, cell death, innate and adaptive immunity, especially in tumor initiation and progress [32–35]. Previous studies have indicated that autophagy plays a role of double-edged sword in disease and health, however in tumor therapy it usually serves as cytoprotective function [32, 36, 37]. Cytoprotective autophagy could be induced by chemotherapeutics such as temozolomide [38–40], Vismodegib [41] and cisplatin [42], and depletion of autophagy could prominently enhance the cytotoxicity of these chemotherapeutics. Importantly, in amino acid deprivation therapy for hematologic and solid malignancies using asparaginase and arginase, autophagy can initiate therapy-resistance [21, 26, 28, 29]. Therefore, we highly speculated that asparaginase could trigger autophagy in GBM cells, and suppression of autophagy could potentiate the anti-tumor effect of asparagine depletion on glioblastoma.

In this study, we found that, apart from growth inhibition and caspase-dependent apoptosis, asparaginase did induce autophagic response in GBM cells. Most importantly, suppression of autophagy by CQ and LY294002 enhanced asparaginase-induced cytotoxic effect and apoptosis. We also determined the mechanism by which asparaginase initiated autophagy. In addition, in tumor xenograft model using U87MG cell line, the combination treatment of asparaginase and CQ significantly reduced the tumor volume and tumor weight. With our findings mentioned above, autophagy plays a cytoprotective role in asparagine deprivation therapy and autophagy abolishment can increase the sensitivity of GMB cells to asparaginase.

## RESULTS

### Asparaginase induced cytotoxicity and apoptosis in GBM cells *in vitro*

**Figure 1:**
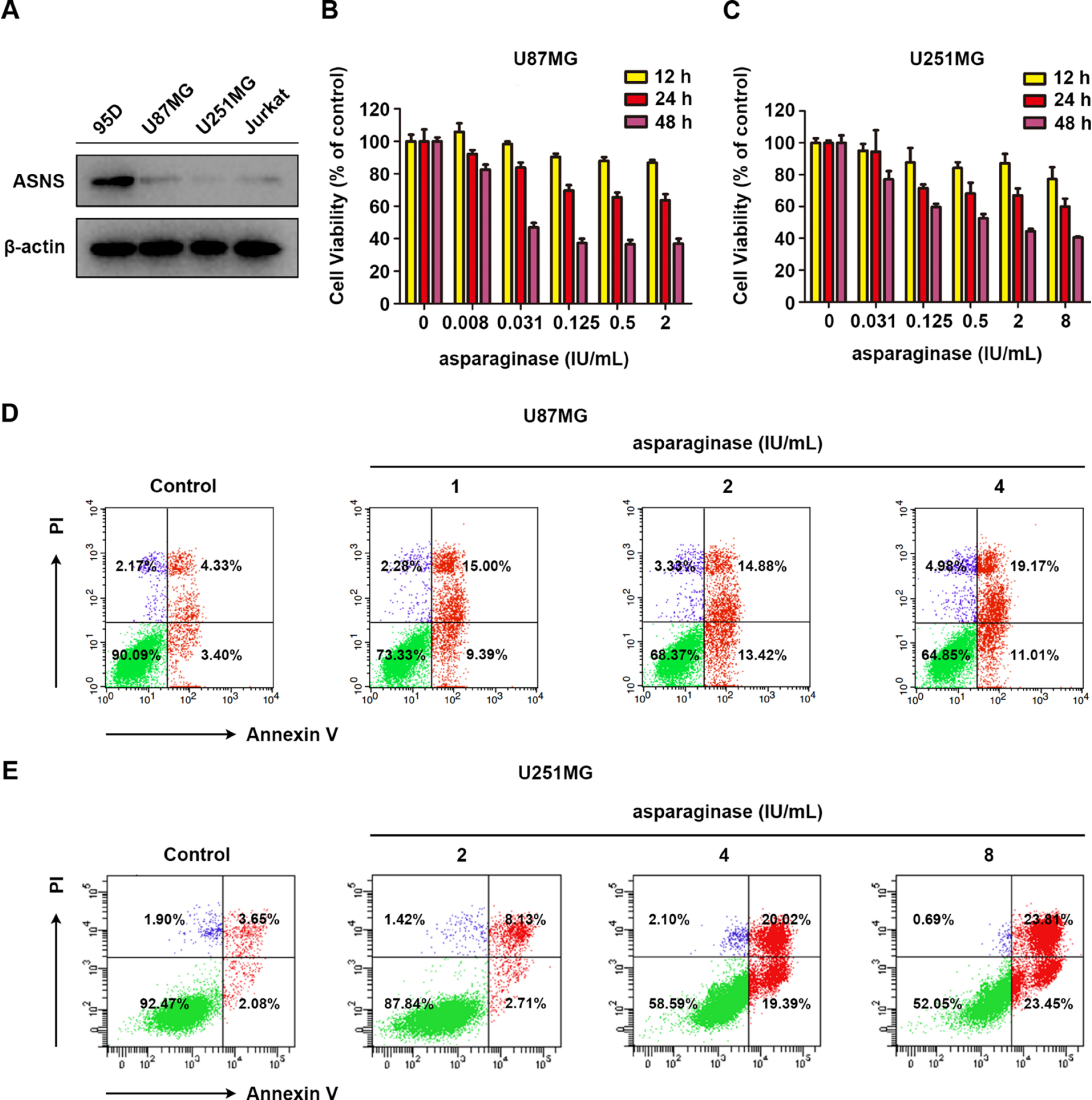
Asparaginase induced cytotoxicity and apoptosis in GBM cells *in vitro* (**A**) Cells were collected and lysed, then western blot analysis was performed to assess the expression level of ASNS in U87MG and U251MG cells. 95D cells were used as a positive control, while Jurkat cells were used as a negative one. (**B**, **C**) U87MG and U251MG cells were incubated with different concentrations of asparaginase for 12, 24 and 48 h, then cell viability was measured by MTT assay. (**D**) U87MG cells were treated with 1, 2, 4 IU/mL of asparaginase for 48 h, and stained with Annexin V/PI, then analyzed by flow cytometry. (**E**) U251MG cells were treated with 2, 4, 8 IU/mL of asparaginase for 48 h, and stained with Annexin V/PI, then analyzed by flow cytometry.

Since asparagine synthetase (ASNS) can catalyze the biosynthesis of L-asparagine by utilizing L-aspartate and L-glutamine as substrate, ASNS-deficient tumor cells were reported to be more sensitive to asparagine deprivation therapy [44, 45]. Herein, western blot analysis was performed to evaluate the expression of ASNS in U87MG and U251MG cells and the results indicated that both GBM cells were ASNS-deficient (Figure 1A). Human highly metastasis lung cancer cell line 95D, which showed drug resistance to asparaginase (Supplementary Figure 1), served as ASNS-positive control [46] and human acute lymphocytic leukemia cell line Jurkat served as ASNS-negative control [47]. Therefore, we supposed that U87MG and U251MG cells were sensitive to asparagine deprivation therapy.

Subsequently, the viability of U87MG and U251MG cells in response to asparaginase was assessed by MTT cytotoxicity assay. As shown in Figure 1B and 1C, prominent cytotoxicity of asparaginase was observed in a dose- and time-dependent manner in U87MG and U251MG cells. Moreover, the percentage of apoptotic cells was determined by FACS analysis. After incubation with various concentrations of asparaginase, the apoptotic rate of U87MG and U251MG cells significantly increased (Figure 1D and 1E), indicating that apoptosis was initiated by asparagine depletion in these two cells.

These results demonstrated that cytotoxicity and apoptosis was induced by asparaginase in U87MG and U251MG GBM cells *in vitro*.

### Asparaginase-induced apoptosis was related to caspase 3 activation in GBM cells

**Figure 2:**
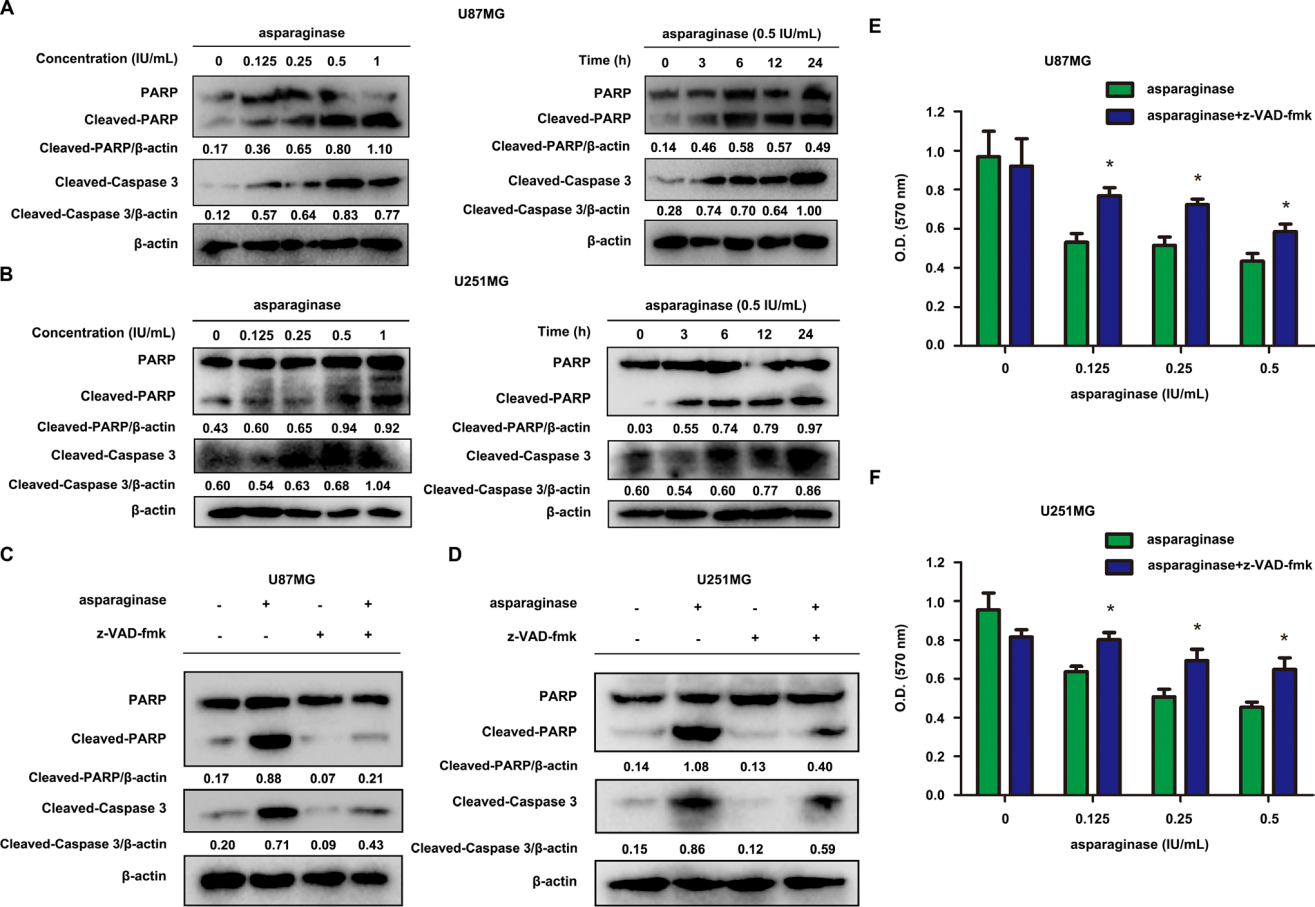
Asparaginase-induced apoptosis was related to caspase 3 activation in GBM cells (**A**, **B**) U87MG and U251MG cells were dose- and time-dependently treated with asparaginase, then western blot analysis was performed to assess the expression level of PARP, cleaved PARP and cleaved-caspase 3. (**C**, **D**) U87MG and U251MG cells were incubated with 0.5 IU/mL of asparaginase, either alone or in combination with 20 μM z-VAD-fmk for 24 h, then western blot analysis was performed to assess the level of PARP, cleaved PARP and cleaved-caspase 3. (**E**, **F**) U87MG and U251MG cells were treated with asparaginase at indicated concentrations in the absence or presence of 20 μM z-VAD-fmk for 24 h, then cell viability was determined by MTT assay at the wavelength of 570 nm. Results were represented as mean ± SD (*P < 0.05).

Generally, the initiation of apoptosis is accompanied by the activation of caspase, especially caspase 3 [48]. We investigated whether asparaginase induced GBM cell apoptosis through caspase-dependent pathway. As shown by western blot analysis, the level of cleaved-PARP and cleaved-caspase 3 increased in both dose- and time-dependent manner following asparaginase treatment (Figure 2A and 2B). In order to further assess the function of caspases in asparaginase-induced apoptosis, U87MG and U251MG cells were exposed to z-VAD-fmk, a pan-caspase inhibitor, together with asparaginase. The western blot analysis showed that the level of both cleaved-PARP and cleaved-caspase 3 markedly decreased when asparaginase was combined with z-VAD-fmk (Figure 2C and 2D). Besides, cytotoxicity assays proved that z-VAD-fmk significantly relieved the growth inhibition of U87MG and U251MG cells induced by asparaginase (Figure 2E and 2F).

Collectively, the results strongly suggested that the apoptosis induced by asparaginase was correlated with the activation of caspase 3 in GBM cells.

### Asparaginase activated autophagy in GBM cells

**Figure 3:**
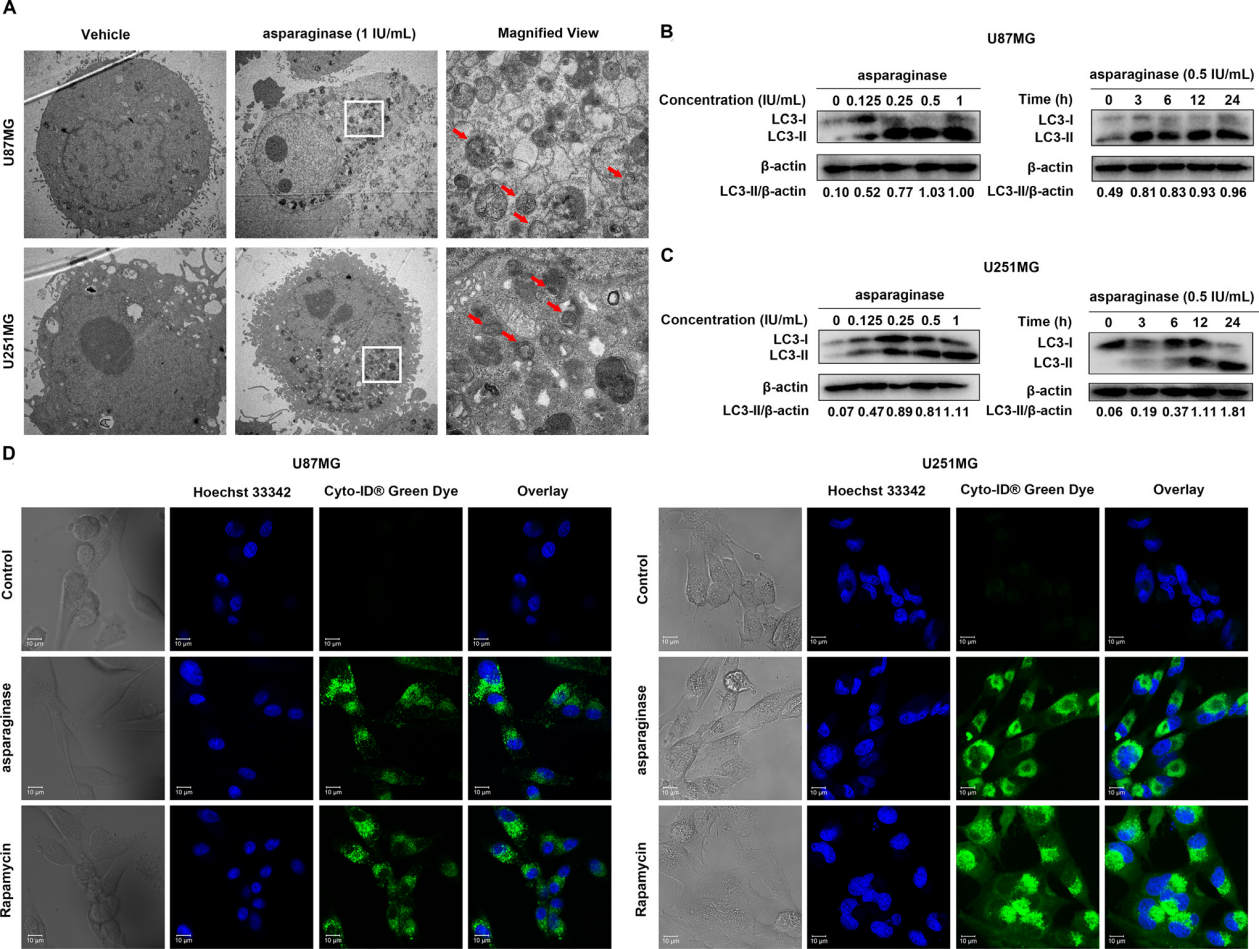
Asparaginase activated autophagy in GBM cells (**A**) U87MG and U251MG cells were treated with 0.5 IU/mL of asparaginase for 24 h. Representative electron micrographs of U87MG and U251MG cells were taken at × 5,000 (left and middle) or × 20,000 (right). (**B**, **C**) U87MG and U251MG cells were dose- and time-dependently treated with asparaginase, then detected autophagy-associate protein LC3-I/II by western blot analysis. (**D**) U87MG and U251MG cells were treated with 0.5 IU/mL of asparaginase for 48 h, then cells were stained with Cyto-ID^®^ Green autophagy dye and examined by confocal fluorescent microscopy.

Whether autophagy participated in asparagine deprivation therapy for glioblastoma was still left to be clarified. Thus, three standard methods including transmission electron microscope (TEM), confocal immunofluorescence and western blot analysis were employed to detect autophagy induction in asparaginase-treated GBM cells. First, TEM was used to observe the autophagosomes formation and accumulation. After 24-hour asparaginase incubation, both U87MG and U251MG cells showed a strong accumulation of double-membrane autophagosomes, while cells in control group showed no obvious occurrence of autophagosomes (Figure 3A). Next, we used western blot analysis to assess the expression of cytoplasmic LC3-I and autophagosome-associated LC3-II. From Figure 3B and 3C, we observed that treatment with asparaginase could induce the conversion of LC3-I to LC3-II dose- and time-dependently. To further confirm the asparaginase-induced autophagy, Cyto-ID^®^ Green Dye which can specifically label autophagic vacuoles was used to stain cells, and fluorescence microscopy was employed to observe the intensity of green fluorescence. The observations showed that U87MG and U251MG cells treated with asparaginase exhibited stronger intensity of green fluorescence than control cells, similar to rapamycin-treated positive control (Figure 3D).

Taken together, these data suggested that autophagy was induced by asparagine depletion in GBM cells.

### Abolishing autophagy potentiated asparaginase-induced growth inhibition and apoptosis of GBM cells *in vitro*

**Figure 4:**
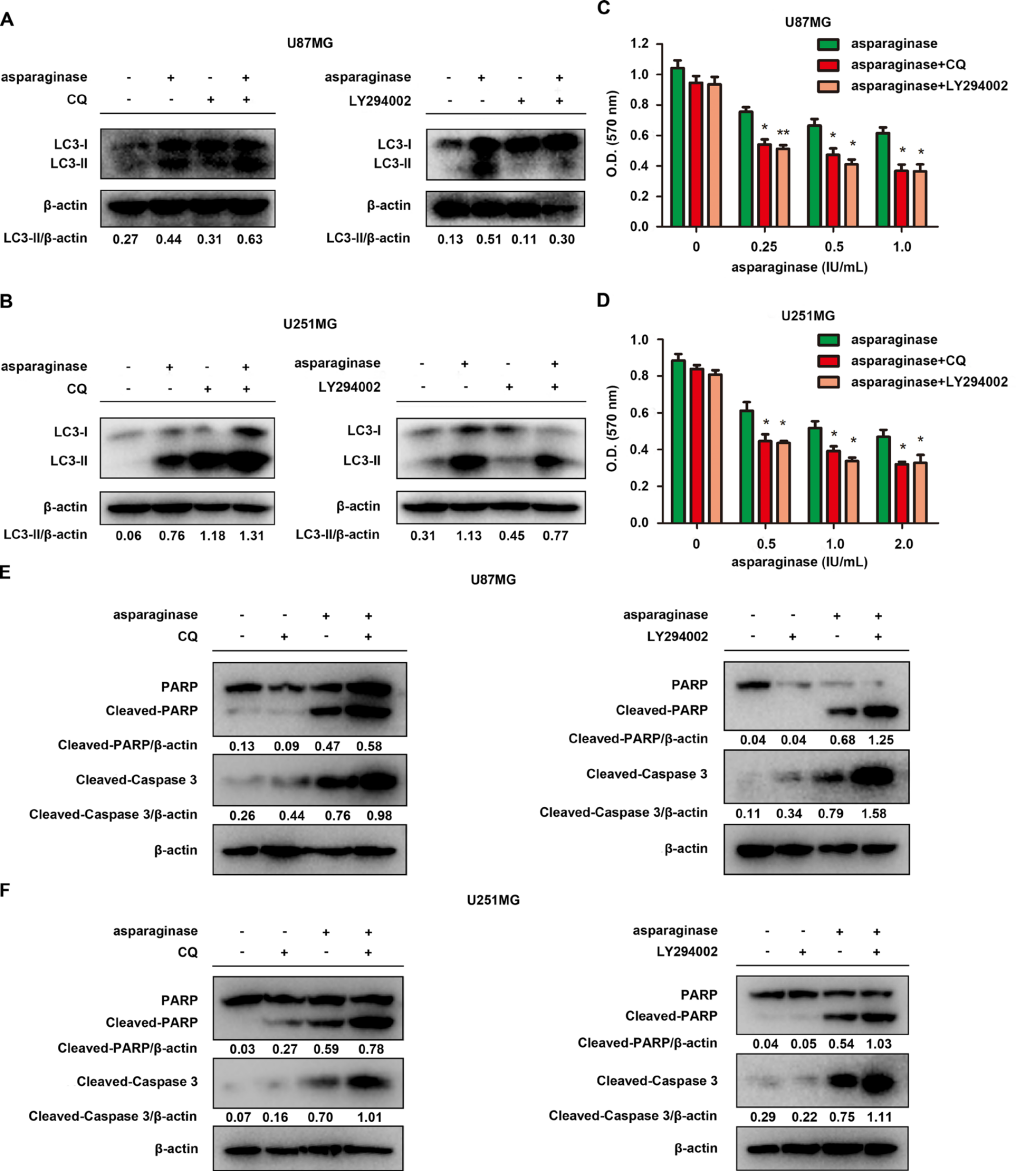
Abolishing autophagy potentiated asparaginase-induced growth inhibition and apoptosis of GBM cells *in vitro* (**A**) U87MG cells were treated with 0.5 IU/mL of asparaginase in the absence or presence of 20 μM CQ or 20 μM LY294002 for 48 h, autophagy-associated protein LC3-I/II were detected by western blot analysis. (**B**) U251MG cells were treated with 0.5 IU/mL of asparaginase in the absence or presence of 20 μM CQ or 10 μM LY294002 for 48 h, autophagy-associated protein LC3-I/II were detected by western blot analysis. (**C**) U87MG cells were treated with asparaginase at indicated concentrations in the absence or presence of 20 μM CQ or 20 μM LY294002 for 48 h, then cell viability was analyzed by MTT assay. (**D**) U251MG cells were treated with asparaginase at indicated concentrations in the absence or presence of 20 μM CQ or 10 μM LY294002 for 48 h, then cell viability was analyzed by MTT assay. (**E**) U87MG cells were treated with 0.5 IU/mL of asparaginase in the absence or presence of 20 μM CQ or 20 μM LY294002 for 48 h, then western blot analysis was performed to assess the expression level of PARP, cleaved PARP and cleaved-caspase 3. (**F**) U251MG cells were treated with 0.5 IU/mL of asparaginase in the absence or presence of 20 μM CQ or 10 μM LY294002 for 48 h, then western blot analysis was performed to assess the expression level of PARP, cleaved PARP and cleaved-caspase 3. Results were represented as mean ± SD (*P < 0.05, **P < 0.01).

To investigate the role of autophagy in asparagine deprivation therapy for glioblastoma, two autophagy inhibitors, chloroquine (CQ) and LY294002, were applied to inhibit autophagy activated by asparaginase. CQ, a lysosome inhibitor blocking the fusion of autophagosomes and lysosomes, can result in overexpression of LC3-II. While LY294002, a strong PI3K inhibitor, can inhibit the accumulation of autophagosomes and the conversion of LC3-I to LC3-II. Western blot analysis demonstrated that both CQ and LY294002 successfully inhibited autophagy in asparaginase-treated GBM cells (Figure 4A and 4B). MTT cytotoxicity assays revealed that cell viability of U87MG and U251MG remarkably decreased when co-treated with asparaginase and either of the autophagy inhibitors, compared with cells treated with asparaginase alone (Figure 4C and 4D). To further understand the biological role of autophagy in asparaginase-induced cell death, we evaluated the apoptosis changes triggered by autophagy inhibition. The western blot analysis showed that, after suppressing autophagy by CQ or LY294002, the level of both cleaved-PARP and cleaved-caspase 3 increased (Figure 4E and 4F), indicating that caspase-dependent apoptosis was markedly encouraged.

These experiments indicated that suppression of autophagy enhanced asparaginase-induced cytotoxicity and apoptosis, suggesting the cellular protective role of autophagy in asparaginase-treated GBM cells.

### The Akt/mTOR and Erk signaling pathway participated in asparaginase-induced autophagy in GBM cells

**Figure 5:**
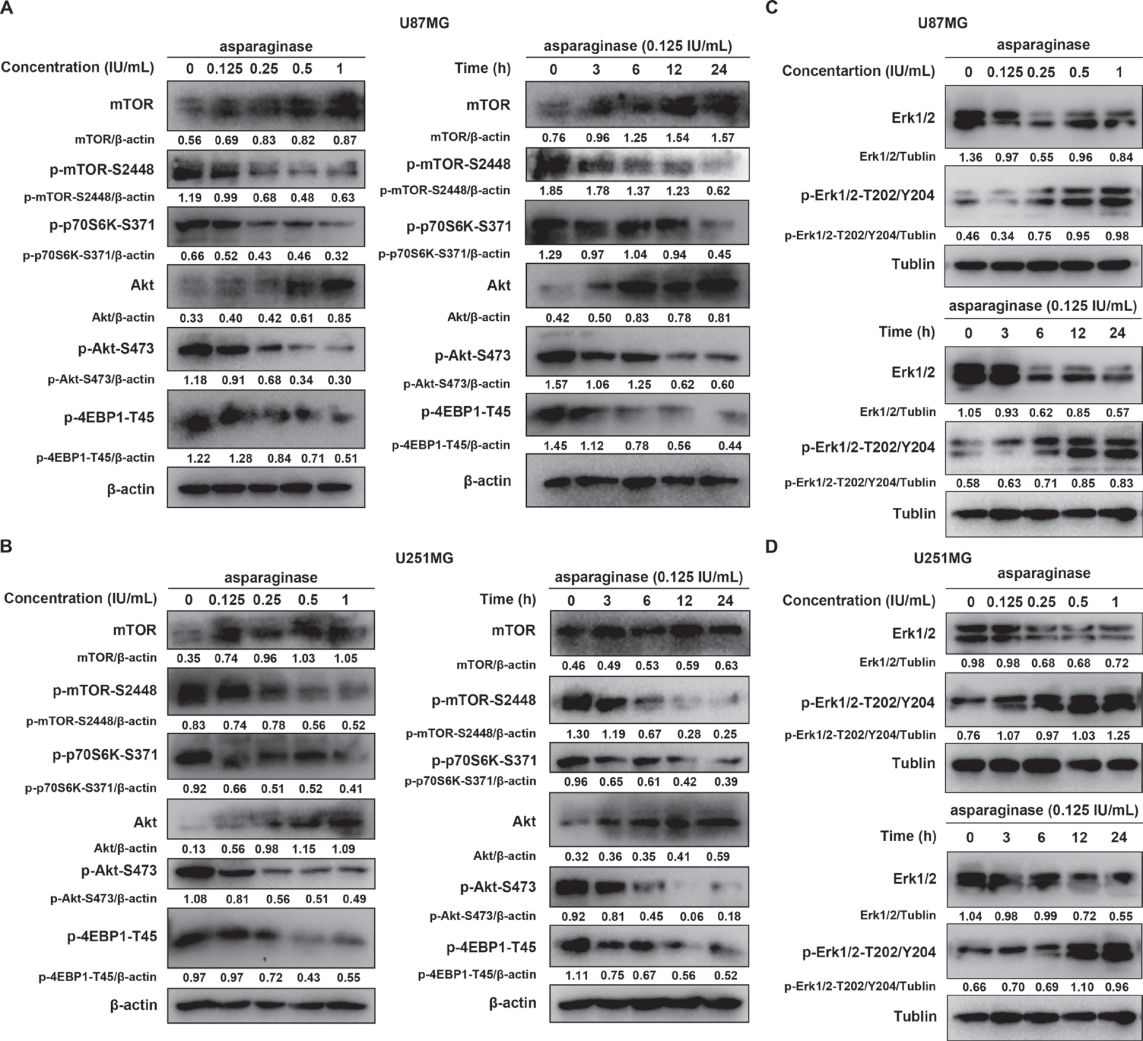
The Akt/mTOR and Erk signaling pathway participated asparaginase-induced autophagy in GBM cells (**A**, **B**) U87MG and U251MG cells were dose- and time-dependently treated with asparaginase, the level of mTOR, p-mTOR, p-p70S6K, Akt, p-Akt and p-4EBP1 were analyzed by western blot analysis. (**C**, **D**) U87MG and U251MG cells were dose- and time-dependently treated with asparaginase, the level of Erk1/2 and p-Erk1/2 were analyzed by western blot analysis.

To uncover the molecular mechanism underlying asparaginase-induced autophagy, we next investigated signaling pathway related to autophagy and the activation status of the key members. The Akt/mTOR signaling pathway is an important signaling pathways negatively regulating autophagy [49]. Nutrient starvation can induce autophagy in eukaryotic cells through inhibition of Akt/mTOR signaling pathway. As shown in Figure 5A and 5B, Akt phosphorylation was significantly reduced in U87MG and U251MG in a dose- and time-dependent manner after treatment with asparaginase, along with the dephosphorylation of mTOR, an essential downstream effector. Besides, the phosphorylation of two downstream substrates, protein S6 Kinase (p70S6K) and eukaryotic initiation factor 4E-binding protein 1 (4EBP-1), was also efficiently suppressed (Figure 5A and 5B).

Extracellular signal-regulated kinase (Erk1/2) is another important pathway involved in autophagy [50]. Then we evaluate the expression of phosphorylated Erk1/2 using western blot analysis. An increased phosphorylation level of Erk1/2 was observed in a dose- and time-dependent manner in U87MG and U251MG cells treated with various concentrations of asparaginase for 48 h or with 0.125 IU/mL of asparaginase for 3, 6, 12 and 24 h (Figure 5C and 5D).

These observations above suggested that the Akt/mTOR and Erk signaling pathways were involved in asparaginase-induced autophagy in GBM cells.

### ROS was involved in autophagy induced by asparaginase in GBM cells

**Figure 6:**
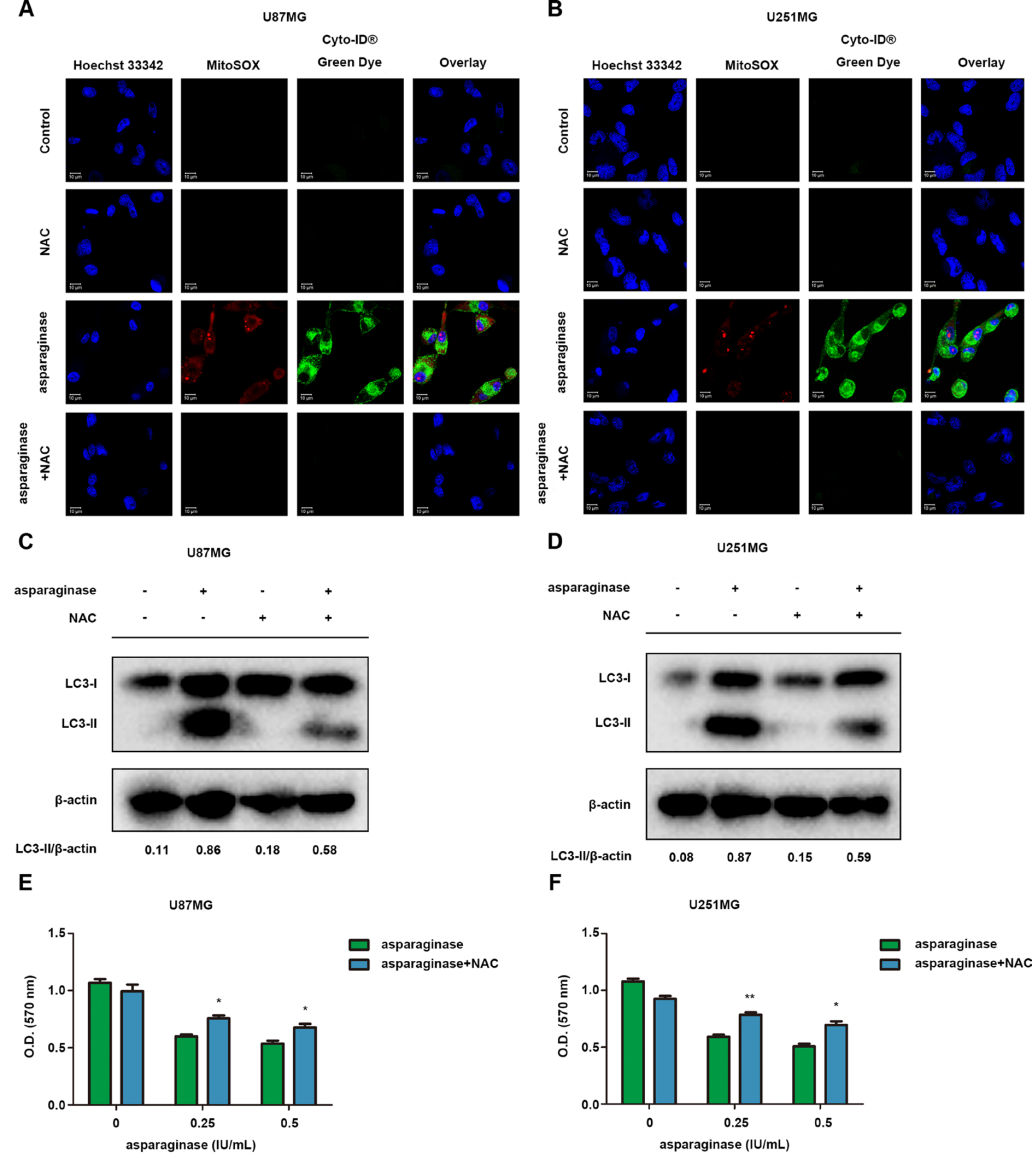
ROS was involved in autophagy by asparaginase in GBM cells (**A**–**D**) U87MG and U251MG cells were treated with 0.5 IU/mL of asparaginase, either alone or in combination with 5 nM NAC for 48 h. (A, B) Cells were stained with MitoSOX and Cyto-ID^®^ Green autophagy dye and examined by confocal fluorescent microscopy. (C, D) Autophagy-associated protein LC3-I/II were detected by western blot analysis. (**E**, **F**) U87MG and U251MG cells were treated with asparaginase at indicated concentrations either alone or in combination with 5 nM NAC for 48 h, then cell viability was determined by MTT assay at the wavelength of 570 nm. Results were represented as mean ± SD (*P < 0.05, **P < 0.01).

ROS accumulation induced by various stresses including nutrient depletion has been confirmed as a critical mediator of autophagy and apoptosis. To investigate whether ROS was involved in asparaginase-induced autophagy in GBM cells, we used ROS detection kit MitoSOX and autophagy detection kit Cyto-ID^®^ to determine intracellular ROS and autophagy generation after cells were exposed to asparaginase. As shown in Figure 6A and 6B, U87MG and U251MG cells treated with asparaginase displayed more mitochondrial ROS-specific red fluorescence and autophagosome-specific green fluorescence than non-treated cells. When cells were pretreated with N-acetyl-cysteine (NAC), a common antioxidant, both red and green fluorescence was remarkably reduced (Figure 6A and 6B). Moreover, western blot analysis revealed that the pretreatment with NAC significantly lowered LC3-II conversion (Figure 6C and 6D), indicating that suppression of ROS generation could block asparaginase-induced autophagy. In addition, scavenging ROS by NAC could rescue GBM cells from the growth inhibition induced by asparaginase (Figure 6E and 6F).

These results revealed that ROS was involved in asparaginase-induced autophagy and cytotoxicity in GBM cells.

### Suppression of autophagy potentiated the anti-glioblastoma effect of asparagine depletion *in vivo*

**Figure 7:**
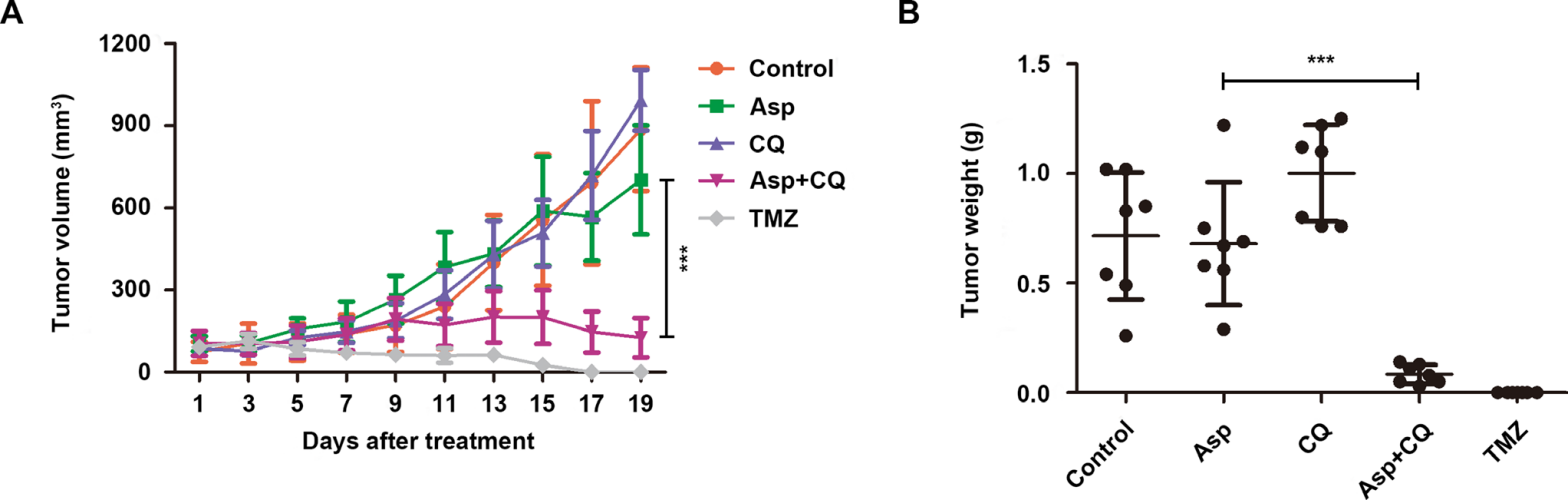
Suppression of autophagy potentiated the anti-tumor effect of asparagine depletion *in vivo* (**A**) Tumor volume changes of U87MG cells xenograft tumor-bearing nude mice. (**B**) Tumor weight of five groups after 19 days of treatment. Results were represented as mean ± SD (***P < 0.001, the tumor volume or tumor weight for the control vs. the tumor volume or tumor weight for the combination of asparaginase and CQ after 19-day treatment).

To examine whether abolishing autophagy potentiates the anti-glioblastoma effect *in vivo*, a subcutaneous xenotransplanted tumor model of human glioblastoma was established using U87MG cell line in nude mice. After tumor formation, the mice were randomized and intraperitoneally injected with sterile isotonic saline, asparaginase, CQ, both agents and temozolomide (positive control). Unlike the prominent growth inhibition *in vitro*, asparaginase alone hardly exhibited anti-tumor effect *in vivo* (Figure 7A and 7B). However, as shown in Figure 7A, the combination treatment with asparaginase and CQ significantly reduced the tumor volume. The average values of tumor volume for the control, asparaginase, CQ, the combination of asparaginase and CQ, and temozolomide after 19-day treatment were 887 mm^3^, 702 mm^3^, 993 mm^3^, 126 mm^3^ and 0, respectively. Moreover, the combination treatment also remarkably reduced the tumor weight (Figure 7B).

In brief, our data revealed that suppression of autophagy potentiated the anti-glioblastoma effect of asparagine depletion *in vivo*.

## DISCUSSION

Glioblastoma is the most common and aggressive primary brain tumor that remains incurable [1]. In spite of progress in the treatment of GBM, the prognosis of patients has remained dismal [3, 4, 8]. Asparaginase has been approved for treatment of childhood ALL by FDA for more than 40 years. Recent studies revealed that asparaginase could be used as a chemotherapeutic agent against not only ALL but also other types of leukemia and several malignant solid cancers [18–26]. In addition, several literature has reported the anti-tumor effect of asparaginase on glioblastoma [10, 11]. However, the anti-glioblastoma effect of asparaginase alone was moderate *in vitro*, and xenograft tumors *in vivo* showed no significant response to asparaginase monotherapy. Besides, previous studies mainly paid attention to synergistic cytotoxicity of asparaginase and other chemotherapeutics, and the underlying mechanism influencing the anti-glioblastoma effect of asparaginase has not been well elucidated.

The present study attempts to reveal the influencing factors in asparagine deprivation therapy for GBM. It has been reported that the tumor cells with particular low ASNS expression were usually sensitive to asparagine depletion, such as primary ALL cells [13], CML cells [51] and several malignant solid cancer cells including ADCA [26], ovarian cancer [25] and hepatocellular carcinoma cells [45]. We first evaluate the level of ASNS expression in GBM cells and found that U87MG and U251MG cells did exhibit a relative low expression of ASNS. Therefore, asparaginase could create a poor asparagine environment for U87MG and U251MG cells. In this study, growth inhibition and apoptosis was observed in asparaginase-treated U87MG and U251MG cells. Further study demonstrated that asparaginase-induced apoptosis was related to caspase 3 activation.

Mounting evidence has indicated that autophagy could be triggered by metabolic stress including amino acid deficiency and served as an adaptive catabolic process [21, 26, 28, 29]. Here, through the formation and accumulation of double-membrane autophagosomes, specially labeled autophagic vacuoles and conversion of LC3-I to LC3-II, we reported for the first time that asparagine deficiency caused by asparaginase could induce autophagy in GBM cells.

Depending on cell type and stimulus, autophagy could be a double-edged sword in cancer treatment—chemotherapeutics could either initiate cytoprotective autophagy or induce autophagic tumor cell death [21, 26, 28, 29, 41, 42, 43]. Most of the time, especially in amino acid deprivation therapy, autophagy could degrade superfluous or dysfunctional proteins and organelles to supply nutrients and materials for dying tumor cells, thus served as protective mechanism [21, 26, 28, 29]. To clarify the role of autophagy in asparagine deprivation therapy for GBM, two autophagy inhibitors with different mechanisms, CQ and LY294002, were adopted to suppress asparaginase-triggered autophagy pharmacologically in U87MG and U251MG cells. We found that suppression of autophagy using both autophagy inhibitors could significantly enhance asparaginase-induced cytotoxicity and apoptosis in U87MG and U251MG cells. These observations strongly indicated the cytoprotective role of autophagy in asparagine deprivation therapy for GBM, suggesting that abolishing autophagy could enhance the anti-glioblastoma effect of asparaginase *in vitro*.

Further research on the mechanism of asparaginase-induced autophagy revealed that Akt/mTOR and Erk signaling pathways were involved in asparagine deprivation therapy for GBM. The Akt/mTOR signaling pathway positively regulates protein translation and negatively regulates autophagy through phosphorylation of Akt, downstream effector mTOR and its substrates p70S6K and 4E-BP1 [49]. Erk signaling pathway plays an important role in almost all cell functions. In response to different stresses, Erk can be activated by active Raf through phosphorylation of threonine and tyrosine residues and induces the formation of cytoplasmic macrovacuoles, a sign of autophagic programmed cell death [50]. Our study showed that, Akt/mTOR signaling pathways were inactivated and Erk signaling pathways were activated, as evidenced by the dose- and time-dependent reduction of Akt, mTOR, p70S6K, and 4EBP-1 phosphorylation, and the dose- and time-dependent increment of Erk phosphorylation.

In general, nutrient deprivation immediately triggers ROS production which irreversibly oxidize DNA and cellular biomolecules, thereby inducing autophagy to maintain metabolic homeostasis. Therefore, ROS accumulation has been confirmed as a critical mediator of autophagy [52, 53]. Herein, we also investigated the role of mitochondrial ROS in the cytotoxicity and autophagy induced by asparaginase in GBM cells. We found that the level of ROS was elevated after cells were treated with asparaginase, and antioxidant NAC could decrease the mitochondrial ROS accumulation. Besides, scavenging ROS by NAC weakened asparaginase-induced cytotoxicity and autophagy. These results revealed that asparaginase-triggered ROS simultaneously initiated cytotoxicity and autophagy in GBM cells.

Finally, to further evaluate the therapeutic efficacy of asparaginase and prove whether suppression of autophagy could potentiate the anti-glioblastoma effect of asparagine depletion *in vivo*, we established a tumor xenograft model using U87MG cell line. Interestingly, although asparaginase could hardly suppress the growth of tumor *in vivo*, which could be explained by drug resistance, the combination treatment with asparaginase and CQ significantly reduced the tumor volume as well as tumor weight, indicating that the drug resistance was overcome by abolishing autophagy. These *in vivo* results indicated the potential therapeutic strategy for GBM by combination of asparaginase and autophagy inhibitors.

**Scheme 1:**
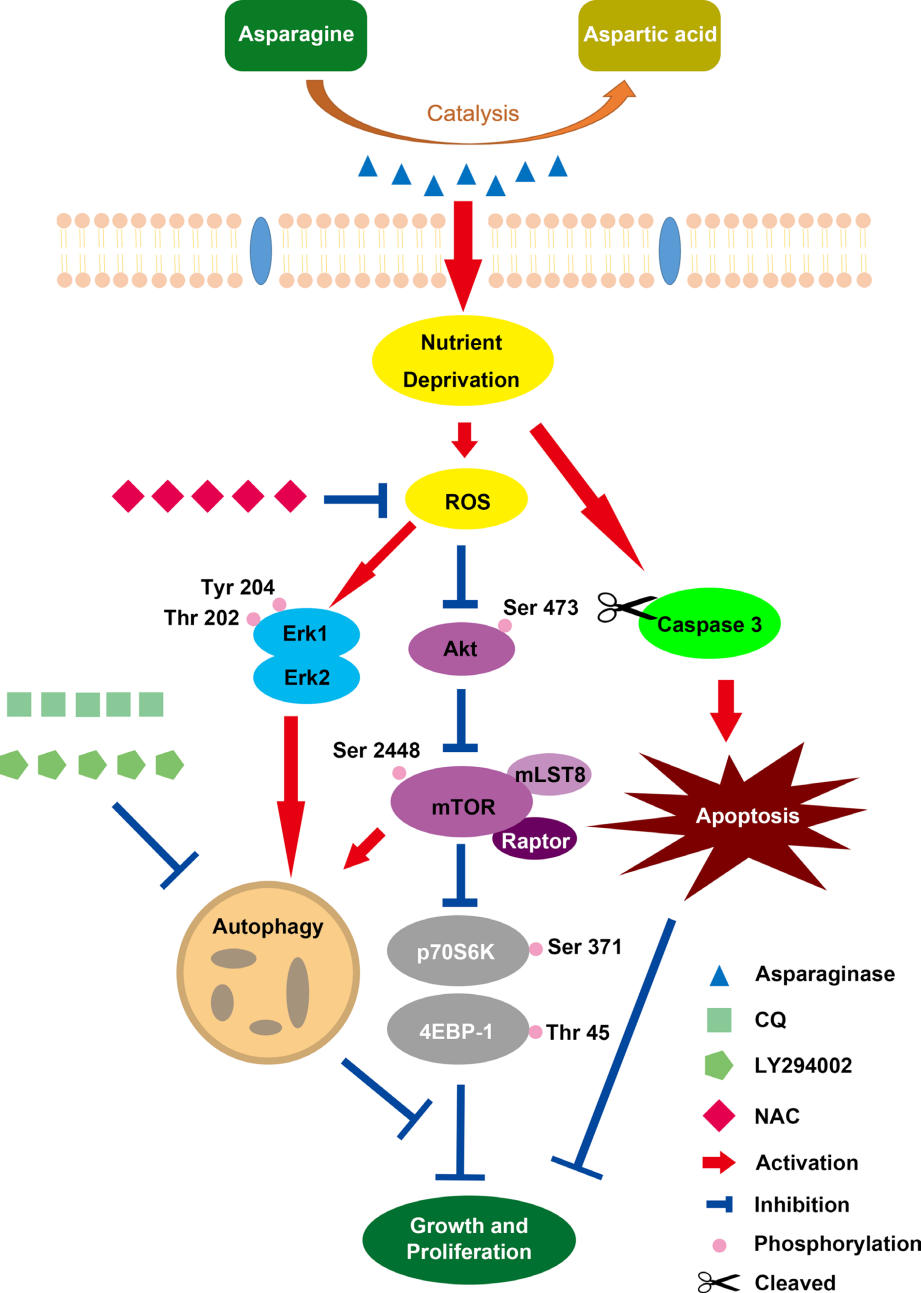
Overview of Caspase-dependent apoptosis and ROS-dependent autophagy and cytotoxicity induced by asparaginase in U87MG and U251MG GBM cells

In conclusion, our present study revealed that asparaginase treatment could induce cytotoxicity and apoptosis in GBM cells. In the meantime, autophagy was initiated by asparagine deficiency. Importantly, inhibition of autophagy could enhance the cytotoxicity and apoptosis of asparaginase *in vitro*, suggesting the cytoprotective role of asparaginase-induced autophagy. Additionally, Akt/mTOR and Erk signaling pathways were proved to be involved in autophagy triggered by asparaginase, and ROS acted as a mediator for both asparaginase-induced cytotoxicity and autophagy (Scheme 1). Also, we demonstrated that suppression of autophagy could potentiate the therapeutic efficacy of asparagine depletion *in vivo*. Therefore, our research highlight that combination of asparagine depletion and autophagy abolishment might be a promising novel therapeutic strategy for glioblastoma.

## MATERIALS AND METHODS

### Cell lines and culture conditions

The human glioma cell lines U87MG and U251MG were purchased from Cell Bank of the Chinese Academy of Sciences, Shanghai Branch (Shanghai, China). Both cells were cultured in Dulbecco’s modified eagle’s medium (DMEM) and supplemented with 10% heat-inactivated fetal bovine serum (FBS), 2 mM L-glutamine, 100 U/mL penicillin and 100 μg/mL streptomycin and incubated at 37°C in a humidified atmosphere of air containing 5% CO_2_. The cells were dissociated using 0.25% trypsin and 0.02% EDTA solution and subcultured routinely once in 2–3 days.

### Reagents and antibodies

Asparaginase (derived from Erwinia) was purchased from Baiyunshan Mingxing Pharmaceutical Co., Ltd. (Guangzhou, Guangdong Province, China). The lysosomal inhibitor chloroquine (CQ) was obtained from Sigma-Aldrich (St. Louis, MO, USA) while the other autophagy inhibitor, the PI3K inhibibtor LY294002 was obtained from Beyotime Institute of Biotechnology (Haimen, Jiangsu Province, China). The autophagy inducer Rapamycin, the pan-caspase inhibitor z-VAD-fmk and ROS scavenger N-avetyl-L-cysteine (NAC) were purchased from Beyotime Institute of Biotechnology (Haimen, Jiangsu Province, China). 3-(4,5-dimetrylthiazol-2-yl)-2,5-diphenyltetrazolium bromide (MTT) was obtained from Sigma-Aldrich (St. Louis, MO, USA). Temozolomide (TMZ) was purchased from Aladdin Industrial Corporation (Shanghai, China). Fluorescein (FITC)-Annexin V Apoptosis Detection Kit was purchased from BD Biosciences (San Diego, CA, USA). Cyto-ID^®^ Green Dye was purchased from ENZO Life Sciences, Inc. (Farmingdale, NY, USA). Primary antibodies including anti-LC3B, anti-PARP, anti-Caspase 3, anti-mTOR, anti-Phospho-mTOR (Ser2448), anti-Phospho-p70 S6 Kinase (Ser371), anti-Akt1, anti-Phospho-Akt (Ser473), anti-4EBP1 Phospho (pT45), anti-p44/42 MAPK (Erk1/2), anti-Phospho-p44/42 MAPK (Erk1/2) (Thr202/Tyr204), anti-Tublin and anti-β-actin were provided by Cell Signaling Technology (Danvers, MA, USA). The secondary antibodies horseradish peroxidase (HRP)-conjugated goat anti-mouse and anti-rabbit immunoglobulin G were provided by MR Biotech (Shanghai, China).

### Cell viability assay

Cell viability was evaluated using 3-(4,5-dimetrylthiazol-2-yl)-2,5-diphenyltetrazolium bromide (MTT) cytotoxicity assay. Briefly, cells in the logarithmic growth phase were seeded in 96-well flat bottom plates at a density of 1 × 10^4^ cells/mL and left to attach overnight prior treatment, then different concentrations of asparaginase with or without autophagy inhibitors were added and compared with DMEM-treated control. Following the 48-hour-treatment, cells were incubated with 0.5 mg/mL of MTT for another 4 h in a humidified incubator at 37°C, then the supernatants were removed and replaced by 100 μl of dimethyl sulfoxide (DMSO). After the formed formazan crystals were completely dissolved, the optical density (O.D.) at the wavelength of 570 nm was measured using a microplate reader. Cell viability was expressed as the ratio of O.D. of treated cells relative to the untreated cells.

### Western blot analysis

To determine the levels of protein expression, U87MG and U251MG cells were harvested and gently washed with cold phosphate-buffered saline (PBS). Then cells were lysed in RIPA Cell Lysis Buffer (Beyotime Institute of Biotechnology, Haimen, Jiangsu Province, China) for at least 30 min at 0°C, and centrifuged at 12,000 at 4°C for 10 min. Supernatants were collected, and the total protein concentration was quantified using the bicinchoninic acid (BCA) assay kit (Biotechwell Biological Technology Co., Ltd, Shanghai, China). Equal amounts of proteins (20 μg) were separated by sodium dodecyl sulfate-polyacrylamide gel electrophoresis (SDS-PAGE) and transferred to polyvinylidene fluoride (PVDF) membranes. After blocking with Tris-buffered saline and Tween 20 (TBST) containing 3% bovine serum albumin (BSA) at room temperature for 2 h, the membranes were incubated with specialized primary antibodies overnight at 4°C, equal lane loading was confirmed using anti-β-actin antibody. Then membranes were washed with TBST buffer for 3 times following the incubation, and incubated with peroxidase-conjugated secondary antibodies for 2 h at room temperature subsequently. After washing with TBST buffer for 3 times, the membranes were scanned using an enhanced chemiluminescent detection kit (Pierce, Rockford, IL, USA).

### Cell apoptosis detected by flow cytometry

Annexin V-fluorescein isothiocyanate (FITC)/propidiumiodide (PI) apoptosis detection kit (BD Biosciences, San Diego, USA) was used to assess apoptotic cells by flow cytometry following manufacturer’s guidelines. In brief, U87MG and U251MG cells were incubated in 6-well flat bottom plates at a density of 2 × 10^5^ cells/well. After treatment, cells were harvested and washed twice using cold PBS. Subsequently, cells were re-suspended in 1× binding buffer at a density of 1 × 10^6^ cells/mL. Then, the cells were incubated with Annexin V-FITC/PI for at least 15 min in an incubator at 37°C. The analysis was performed using a FACS Calibur flow cytometer. Both Annexin V^+^/PI^+^ and Annexin V^+^/PI^−^cells were considered as apoptotic cells.

### Transmission electron microscopy

After being incubated with or without asparaginase for 24 h, U87MG and U251MG cells were collected and fixed with 2% ice-cold glutaraldehyde. Then samples were analyzed with a JEM 1400 transmission electron microscopy (JEOL, Inc., USA) at 80 kV.

### Confocal immunofluorescence

U87MG and U251MG cells were seeded in cell culture dishes with glass bottoms (NEST Biotechnology, Jiangsu Province, China), then treated with or without asparaginase (1 IU/mL) for 48 h after complete attachment. Cells treated with autophagy inducer rapamycin (50 mM) for 2 h were served as positive controls. Subsequently, cells were stained with Cyto-ID^®^ Green dye and Hoechst 33342 for 15 min at 37°C and observed using a confocal microscope (Carl Zeiss LSM710, Carl Zeiss, Germany). All the steps were performed in the dark.

### Tumor xenograft model

To evaluate the anti-glioma effect of asparaginase alone or in combination with autophagy inhibitor chloroquine (CQ) *in vivo*, a xenograft model of human glioma was established. Female BALB/c nude mice (18 ± 2 g) were purchased from Experimental Animal Center of Fudan University and raised under standard housing condition. All mouse experiments were complied with guidelines of the Animal Experimentation Ethics Committee of Fudan University. After 1-week acclimatization, each mouse was injected under the skin in the right flank with 5 × 10^6^ U87MG cells re-suspended in 100 μl DMEM. On day 7 after transplantation, when the tumors reached an average size of 100 to 150 mm^3^, the mice (*n* = 35) were randomly divided into 5 groups (*n* = 7/group): control group (sterile isotonic saline), asparaginase group (asparaginase 5 IU/g), CQ group (0.05 mg/g), asparaginase + CQ group (asparaginase 5 IU/g + CQ 0.05 mg/g) and positive control group (temozolomide 0.02 mg/g). Asparaginase was injected intraperitoneally every day, while CQ and temozolomide were injected intraperitoneally every 2 days. Tumor diameter was measured every 2 days throughout the experiment with digital calipers and an ellipsoid volume formula was used to calculate tumor volumes (1/2 × length × width^2^). Mice were euthanized when tumor volume reached about 1,000 mm^3^, then tumors were carefully removed and weighed.

### Statistical analysis

Data were expressed as mean ± standard deviations (SD). The statistical significance of the differences between groups was analyzed using Student’s *t* test. *, ** and *** indicated *P* < 0.05, *P* < 0.01 and *P* < 0.001, respectively.

## SUPPLEMENTARY MATERIALS FIGURE


