# Supplementary material for: Autophagy suppression potentiates the anti-glioblastoma effect of asparaginase in vitro and in vivo
Source: Oncotarget. 2017 Jul 20;8(53):91052–66. doi: 10.18632/oncotarget.19409 (PMC5710905; doi:10.18632/oncotarget.19409)
Supplement: Supplementary file 1 [file oncotarget-08-91052-s001.pdf]

Autophagy suppression potentiates the anti-glioblastoma effect of asparaginase *in vitro* and *in vivo*

SUPPLEMENTARY MATERIALS

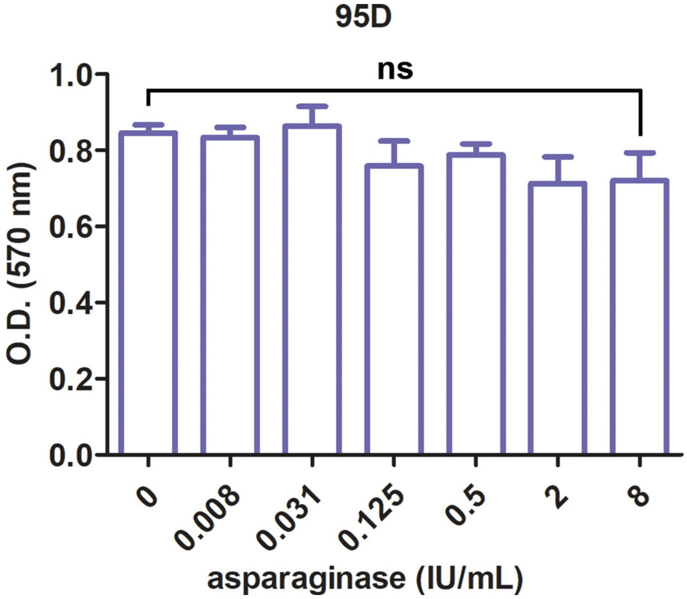

Supplementary Figure 1: Human highly metastasis lung cancer cell line 95D showed drug resistance to asparaginase. 95D cells were incubated with different concentrations of asparaginase for 48 h, then cell viability was measured by MTT assay.
